# A Critical Function for the Transcription Factors GLI1 and GLI2 in the Proliferation and Survival of Human Mast Cells

**DOI:** 10.3389/fimmu.2022.841045

**Published:** 2022-02-16

**Authors:** Guido Hernan Falduto, Annika Pfeiffer, Qunshu Zhang, Yuzhi Yin, Dean Darrel Metcalfe, Ana Olivera

**Affiliations:** Mast Cell Biology Section, Laboratory of Allergic Diseases, National Institute of Allergy and Infectious Diseases, National Institutes of Health, Bethesda, MD, United States

**Keywords:** mast cell, GLI, hedgehog signaling pathway, KIT, apoptosis, proliferation

## Abstract

Mast cell hyperactivity and accumulation in tissues are associated with allergy and other mast cell-related disorders. However, the molecular pathways regulating mast cell survival in homeostasis and disease are not completely understood. As glioma-associated oncogene (GLI) proteins are involved in both tissue homeostasis and in the hematopoietic system by regulating cell fate decisions, we sought to investigate the role for GLI proteins in the control of proliferation and survival of human mast cells. GLI1 transcripts were present in primary human mast cells and mast cell lines harboring or not activating mutations in the tyrosine kinase receptor KIT (HMC-1.1 and HMC-1.2, and LAD2 cells, respectively), while GLI2 transcripts were only present in HMC-1.1 and HMC-1.2 cells, suggesting a role for oncogenic KIT signaling in the regulation of GLI2. Reduction in GLI activity by small molecule inhibitors, or by shRNA-mediated knockdown of GLI1 or GLI2, led to increases in apoptotic cell death in both cultured human and murine mast cells, and reduced the number of peritoneal mast cells in mice. Although GLI proteins are typically activated *via* the hedgehog pathway, steady-state activation of GLI in mast cells occurred primarily *via* non-canonical pathways. Apoptosis induced by GLI silencing was associated with a downregulation in the expression of KIT and of genes that influence p53 stability and function including USP48, which promotes p53 degradation; and iASPP, which inhibits p53-induced transcription, thus leading to the induction of p53-regulated apoptotic genes. Furthermore, we found that GLI silencing inhibited the proliferation of neoplastic mast cell lines, an effect that was more pronounced in rapidly growing cells. Our findings support the conclusion that GLI1/2 transcription factors are critical regulators of mast cell survival and that their inhibition leads to a significant reduction in the number of mast cells *in vitro* and *in vivo*, even in cells with constitutively active KIT variants. This knowledge can potentially be applicable to reducing mast cell burden in mast cell-related diseases.

## Introduction

Mast cells are immune cells of the myeloid lineage with key roles in the initiation of allergic reactions and in the regulation of chronic inflammation. Activated mast cells mediate these functions through the release of a vast variety of vasoactive and immune-modulatory molecules (1). The severity of mast cell-mediated reactions generally depends on the extent and duration of mast cell responses, and it is influenced by the number of mast cells in tissues (2).

Mast cells terminally differentiate in tissues, where they take up long-term residence. Mast cell numbers are increased in allergic disorders such as asthma, allergic rhinitis, food allergy and atopic dermatitis (2, 3), partly due to enhanced recruitment of mast cell progenitors to the affected sites followed by their maturation (1, 4, 5). Abnormal increases in mast cells are also seen in other mast cell disorders such as mastocytosis, where clonal expansion of mast cells occurs in organs such as the skin, bone marrow and the gastrointestinal tract among others, in association with somatic activating mutations in the tyrosine kinase receptor KIT (CD117) (6, 7). Indeed, KIT enhances the survival, proliferation and function of mast cells and plays an important role in tissue mast cell homeostasis *in vivo* (8) as evidenced also by the lack of tissue mast cells in mice with a deficiency in KIT or its ligand (9), and the observed decrease in mast cell counts after blocking KIT activity in humans (10, 11). However, the regulatory mechanisms balancing mast cell survival (via KIT or other microenvironmental signals) and cell death to maintain mast cell homeostasis in health and disease are not well understood. Knowledge of these mechanisms may be relevant for strategies to reduce mast cells numbers and activity in mast cell-related disorders (2, 12, 13).

**Figure 1:**
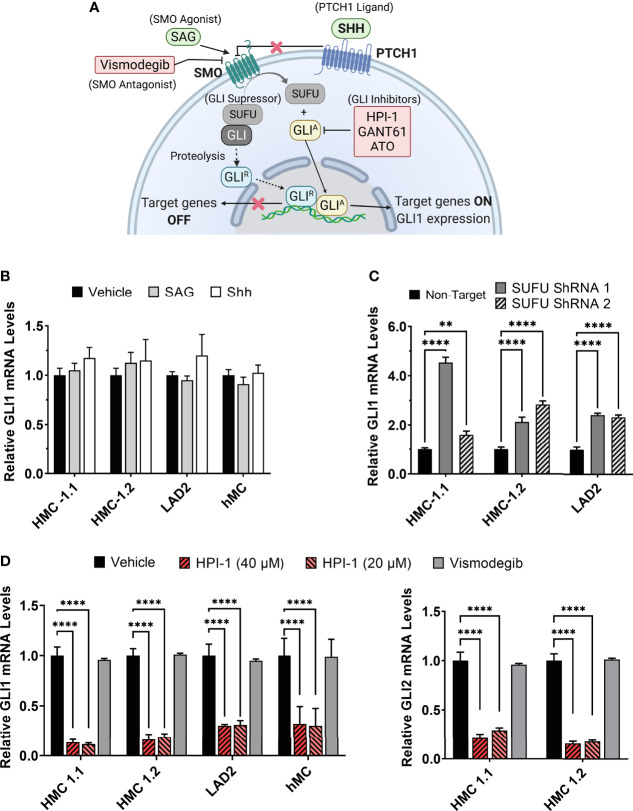
Modulation of GLI1/2 expression in human mast cells. **(A)** Simplified illustration of the canonical hedgehog signaling pathway and some of the small molecules used for its activation or inhibition. **(B)** Relative GLI1 mRNA levels (2^-ΔΔCq^) in human mast cell lines (HMC-1.1, HMC-1.2, LAD2) and primary human mast cells (hMC) after 20 h incubation with the SMO agonist SAG (200 nM) or the PTCH1 ligand SHH (500 ng/mL). **(C)** Relative GLI1 mRNA levels (2^-ΔΔCq^) in mast cells transduced with lentiviral particles containing two separate shRNA constructs to knockdown SUFU (see **Supplementary Figure 2**). **(D)** Relative GLI1 and GLI2 mRNA levels (2^-ΔΔCq^) after 5 h culture with the GLI1/2 inhibitor HPI-1 (20 or 40 µM) or with the SMO inhibitor vismodegib (40 µM). Results are expressed as Mean ± SD of three independent experiments. GAPDH and ACTB were used for normalization. Each individual experiment was done in triplicate. Two-way ANOVA followed by Dunnet multiple comparisons test was used for statistical analysis. **p < 0.01; ****p < 0.0001.

Glioma-associated oncogene family (GLI) transcription factors regulate the expression of various genes critical for proliferation, survival, genetic stability, and cell determination, during embryonic development and later in life. One of these target genes is *KIT*, whose promoter region contains two GLI consensus-binding sites (14) and its expression can be negatively or positively regulated depending on the GLI isoforms (14–16). GLI transcription factors consist of three family members, namely GLI1, GLI2 and GLI3. The canonical pathway for the activation of GLI is the hedgehog pathway (HH), although increasing evidence demonstrates that transcriptional expression and post-translational modifications leading to the activation of GLI also occur by non-canonical mechanisms (17). In the canonical HH-pathway (**Figure 1A**), the 12-pass transmembrane receptor Patched 1 (PTCH1) is activated by the HH ligands sonic (SHH), Indian (IHH), and desert HH (DHH). In their absence, PTCH1 inhibits the 7-pass transmembrane G protein-coupled receptor smoothened (SMO), leading to the partial proteasomal degradation of GLI3 and to a much lesser extent of GLI2, to form transcriptional repressor forms (GLI^R^). In the presence of HH ligands, PTCH1 stops the inhibition of SMO, proteolysis of GLI is blocked and thus active, full length GLI activator forms (GLI^A^) accumulate and translocate into the nucleus to induce gene transcription. Unlike GLI2 and GLI3, GLI1 is not subject to proteolysis, and its levels are mainly regulated by induction of transcription, which is the reason why GLI1 is used as a reliable reporter of HH activity (18, 19). Overall, GLI1 acts exclusively and GLI2 primarily as transcriptional activators (GLI^A^), while GLI3 acts as the main transcriptional repressor due to its proteolytic processing (20, 21). The GLI proteins are also regulated by sequestration with suppressor of fused (SUFU), which prevents them from entering the nucleus, until activation mechanisms dissociate the complex (**Figure 1A**) (22).

Dysregulation of GLI^A^ and GLI^R^ has been linked to hematological malignancies such as chronic and acute myeloid leukemia among other types of cancer (23, 24). More recently, in a subset of patients with congenital aggressive mastocytosis, a germline mutation causing reduced GLI3^R^ compared to GLI3^A^ was identified, which was associated with mast cell transformation (16). Considering these associations, the role of GLI proteins in cell fate decisions, and their reported regulatory role in KIT expression, we aimed to investigate a potential involvement of GLI transcription factors and the HH-pathway in the proliferation and survival of normal and neoplastic human mast cells. By using inhibitors of GLI and components of the HH-pathway and by shRNA-mediated approaches, we show that homeostatic regulation of GLI1 and GLI2, largely through non-canonical pathways, plays an important role in the survival of human and mouse mast cells *in vitro* and *in vivo*, as well as in the proliferation and survival of neoplastic mast cells carrying or not oncogenic KIT variants. As will be shown, such data provides relevant information on the function of GLI proteins in mast cell biology and tissue homeostasis, and on the molecular underpinnings for this regulation, which includes the regulation of KIT and p53. Furthermore, these additions to the understanding of the molecular pathways that regulate mast cell proliferation, survival and death have relevance in developing novel pharmacological strategies to decrease mast cell numbers, and in the treatment of mast cell-associated disorders.

## Materials and Methods

### Inhibitors and Agonists

The tyrosine kinase inhibitor (TKI) dasatinib, SMO agonist (SAG)- hydrochloride, GLI1/2 inhibitor GANT61, and SMO antagonist vismodegib (GDC-0449) were purchased from Selleckchem (Houston, TX). GLI1/2 inhibitor HPI-1, PI3Kα/δ/β inhibitor LY-294002 hydrochloride, and MEK1/2 inhibitor U0126 were purchased from Tocris (Minneapolis, MN). High activity recombinant Human Sonic Hedgehog (SHH) Protein was purchased from R&D Systems (Minneapolis, MN) and arsenic trioxide (A_2_O_3_ or ATO) from Millipore Sigma (Burlington, MA).

### Animal Studies

Ptch1^+/−^ mice (Ptch1^tm1Mps^/J; Stock No: 003081) were obtained from The Jackson Laboratory (Bar Harbor, ME) (25) and bred to obtain Wt and Ptch1^+/−^ littermates in AAALAC-accredited NIAID animal facilities. C57BL/6J mice were obtained from the Jackson Laboratory and injected every other day with GLI1/2 inhibitors for a total of 7 injections. Mice were randomly divided into three groups (n=4/group) and injected intraperitoneally (150 µL) with either saline vehicle control (PBS : EtOH; 95:5%), HPI-1 (2 mg/kg), or GANT61 (2 mg/kg). Two days after the last injection, mice were euthanized, peritoneal lavage was performed, and mast cell percentages (FcϵRI^+^ Kit^+^ cells) were obtained by FACS analysis. Cells were resuspended at 10^7^ cells/mL in PBS plus LIVE/DEAD™ Fixable Green Dead Cell Stain Kit (Thermo Fisher Scientific, Waltham, MA) according to the manufacturer’s instructions. Cells were then washed, resuspended in FACS buffer (PBS + 3% FBS + 2mM EDTA), and Fcγ receptors blocked with anti-CD16/CD32 (clone 2.4G2, BD Biosciences, Franklin Lakes, NJ). Cells were stained with an optimal amount of anti-Kit-PE (clone ACK2, eBioscience) and anti-FcϵRI-APC (clone MAR-1, Biolegend, San Diego, CA). Data acquisition was performed on a LSRFortessa™ (BD Biosciences) and analyzed using FlowJo software (Tree Star, Ashland, OR). These experiments with live mice were performed under an animal study proposal (LAD2E) approved by the NIAID-DIR-Animal Care and Use Committee, in accordance with federal regulatory requirements and ethical standards under the guidance of the Office of Animal Care and Use of the National Institutes of Health.

### Cell Culture

Mouse bone marrow-derived mast cells (BMMC) were differentiated from the marrow of tibias and femurs of Wt and Ptch1^+/−^ littermate mice and cultured for at 6-8 weeks in RPMI 1640 supplemented with 10% FBS, HEPES (1M), penicillin (100 U/mL), streptomycin, 4 mM L-glutamine (100 μg/mL), sodium pyruvate (1 mM), 2-mercaptoethanol (50 μM), and IL-3 (30 ng/mL, R&D Systems). Cultures were analyzed by flow cytometry and BMMC were identified as FcϵRI and Kit double positive cells as described above. Degranulation assays of BMMC were performed as described (26).

HMC-1.1 and HMC-1.2 cell lines were cultured in Iscove’s Modified Dulbecco’s Medium supplemented with 10% FBS, L-Glutamine (2 mM), penicillin (100 U/ml), streptomycin (100 μg/ml). LAD2 cells were cultured in StemPro-34™ supplemented with StemPro-34™ Nutrient Supplement (Gibco), L-Glutamine (2 mM), penicillin (100 U/ml), streptomycin (100 μg/ml), and SCF (100 ng/ml, R&D Systems).

Primary human mast cell cultures (hMC) were derived from CD34+ lymphocytapheresis progenitors obtained from healthy volunteers following informed consent under a protocol (NCT00001756) approved by the National Institutes of Health Internal Review Board. Cells were cultured as described (27) and used between 7–9 weeks of culture when >95% were mast cells identified as KIT and FcεRI double positive cells.

### Growth Assays

The growth of HMC-1.1 and HMC-1.2 cells was quantified using CyQUANT™ Direct Cell Proliferation Assay (Thermo Fisher Scientific). Cells (3x10^4^) were seeded in 100 µL in 96-well black/clear bottom plates (Corning Inc., Corning, NY) and cultured for 72 h in the presence of inhibitors of GLI or agonists/antagonist of the hedgehog pathway activators as indicated in the figures. Vehicle controls (up to 0.1% DMSO for HPI-1, 0.08% ethanol for GANT61, and PBS for ATO), and control samples containing no vehicle were included in all experiments. These vehicles did not affect growth of the cells. Cells were stained with the Cyquant dye following the manufacturer’s instructions. The relative fluorescence units (RFU) were determined using a Spark^®^ microplate reader (TECAN, Männedorf, Switzerland). For LAD2 cells, 6x10^5^ cells were seeded in 6-well plates (2 mL) and cultured for 7 days in the presence or absence of inhibitors. Viable cells were counted using a Acridine Orange/Propidium Iodide (AO/PI) cell viability kit using a LUNA-FL™ Dual Fluorescence Cell Counter (Logos Biosystems, Gyeonggi-do, South Korea).

### Proliferation Assays

To identify proliferating cells, mast cell lines were stained with 5 µM of Cell Trace Violet (Thermo Fischer Scientific) in PBS following manufacturer’s guidelines. After washing, 5x10^5^ cells were plated in 6 well plates (2 mL/well) with or without the indicated concentrations of inhibitors and cultured for 72 h (HMC-1.1 and HMC-1.2) or 7 days (LAD2) in culture media. To determine viability, cells were collected and stained with a LIVE/DEAD™ Fixable Green Dead Cell Stain Kit (Thermo Fischer Scientific) in PBS for 10 min on ice. Data acquisition was performed on a LSRFortessa™ and analyzed using FlowJo software. After gating out dead cells (LIVE/DEAD™ stained), generations of proliferating cells were recognized by diminishing fluorescence intensity. Cultures of BMMC were stained with 5 µM of Cell Trace Violet (CTV), plated in 6-well plates (5x10^5^ cells in 2 mL) and after 6 days, proliferation and cell viability were analyzed as described above. Cultures of hMC differentiated for 7-9 weeks were plated in 24-well plates (2.5x10^5^ cells in 1 mL) in growth media with or without the indicated concentrations of inhibitors, and cell viability was analyzed after 5 days as described above.

### Apoptosis Assays

To identify cells undergoing apoptosis, expression of surface phosphatidylserine was determined by annexin V staining. Cells (5x10^5^) were cultured in 6-well plates (2 mL) for 48 h (HMC-1.1 and HMC-1.2), 6 days (LAD2), or 4 days (hMC) in the presence or absence of the indicated concentrations of inhibitors and stained using Pacific Blue™ Annexin V/SYTOX™ AADvanced™ Apoptosis Kit (Thermo Fischer Scientific) following the manufacturer’s instructions. Cells were analyzed for annexin V expression in a LSRFortessa™.

In some experiments, apoptosis was further demonstrated by quantifying caspase-3/7 activity using CellEvent™ Caspase-3/7 Green Detection Reagent (Thermo Fischer Scientific), a specific substrate for caspase 3/7 that becomes fluorescent after cleavage. HMC-1.1 or HMC-1.2 cells (5x10^5^) were cultured with or without various treatments for 48 h in 96-well black/clear bottom plates (100 µL), following the manufacturer’s instructions, and RFU were measured in a Spark^®^ microplate reader.

### ShRNA-Mediated Knockdown

Knockdown of GLI1 and GLI2 was conducted by transducing cells with GLI-specific ShRNA constructs. Bacterial glycerol stocks containing the pLKO.1-puro plasmid shRNA constructs were obtained from Millipore Sigma: TRCN0000020486 (construct 1) and TRCN0000232063 (construct 2) for GLI1; and TRCN0000033329 (construct 1) and TRCN0000033330 (construct 2) for GLI2. Plasmid DNA was isolated from individual carbenicillin-resistant colonies using Qiaprep Spin Miniprep Kit (Qiagen). As a non-target control, we used a pLKO.1-puro non-Mammalian shRNA Control Plasmid DNA (SHC002). Lentiviral particles were produced by following the manufacturer’s protocol. Briefly, the packaging lentiviral genes and viral envelop gene (Mission Lentiviral packaging mix, Millipore Sigma) along with plasmid DNA (3.4 µg) were co-transfected into HEK 293T cells (70% confluency in T75 flask) using FuGENE6 transfection reagent (Roche, Indianapolis, IN) to produce lentiviral particles containing the shRNA constructs. The transfected 293T cells were grown in Dulbecco modified Eagle medium that contained FBS (10%) and L-glutamine (4 mM). At 16 h post-transfection, the media was removed and replaced with fresh Dulbecco modified Eagle medium that included penicillin (100 U/mL) and streptomycin (100 mg/mL) and 10% FBS. Supernatants containing viral particles were collected at 40-44 h and 66-70 h post-transfection and combined. Viral particle titer was quantified by using HIV Type 1 p24 Antigen ELISA (ZeptoMetrix Corporation, Buffalo, NY). Cells plated in 6-well plates were transduced with the supernatants containing lentivirus, using approximately 5-10 transduction units (TU)/cell. After 20 h, transduced cells were changed to virus-free medium and selected by adding 2 µg/mL puromycin (*In vivo*gen, San Diego, CA) for HMC-1.2 cells or 0.5 µg/mL puromycin for LAD2 cells. After 5 days of selection, cells were changed to puromycin-free medium and used for experiments.

To knockdown SUFU, lentiviral transduction particles TRCN0000019464 (construct 1), TRCN0000358840 (construct 2) for HMC-1.1 and HMC-1.2, TRCN0000358906 (construct 1), TRCN0000358838 (construct 2) for LAD2, and non-Target ShRNA control were purchased from Millipore Sigma. Cells were transduced as described above, except that no selection was conducted, and RNA was extracted at 96 h post-transduction.

### Quantitative Real-Time PCR

Total RNA from 1-3 × 10^6^ cells, treated as specified in the figure legends, was extracted using RNeasy plus mini kit (Qiagen, Valencia, CA). Reverse transcription and real time qPCR reactions to quantify various gene transcripts were performed in one step using iTaq Universal Probes One-Step Kit (Bio-Rad, Hercules, CA). The following PrimePCR™ probe sets were also purchased from Bio-Rad: SHH (qHsaCEP0040459) PTCH1 and Ptch1 (qHsaCEP0055042 and qMmuCEP0053013), SMO and Smo (qHsaCEP0051485 and qMmuCIP0032784), GLI1 and Gli1 (qHsaCEP0050608 and qMmuCEP0054131), GLI2 (qHsaCEP0057630), GLI3 (qHsaCEP0050421), SUFU (qHsaCEP0058238), KIT (qHsaCIP0026913), PPP1R13L (iASPP) (qHsaCEP0057704), and USP48 (qHsaCEP0049878). GAPDH and Gapdh (qHsaCEP0041396 and qMmuCEP0039581) and ACTB (qHsaCEP0036280) were used as reference genes.

For gene expression profiling of differentially expressed genes within the mitochondrial apoptotic pathway, we used predesigned 96-well panels with probes for 24 genes (Human, Catalog# 10025095, from Bio-Rad). cDNA (25 ng/reaction) obtained from cells after various treatments and using iScript cDNA synthesis Kit (Bio-Rad), were distributed on the plates and amplified using SYBR^®^ Green. All the PCR reactions were conducted in CFX96 Touch Real-Time PCR Detection System (Bio-Rad).

### Western-Blot

Cell lysates were obtained by lysing 4-8x10^6^ cells, treated as specified in the figure legends, in 200-400 µL of RIPA Buffer (Cell Signaling Technologies, Danvers, MA) containing Protease/Phosphatase Inhibitor Cocktail (Roche) for 25 min on ice. Samples were incubated for 10 min at 70°C and separated by electrophoresis on 4–12% NuPage Bis-Tris gels (Thermo Fisher Scientific). Proteins were transferred to nitrocellulose membranes (0.2 μm pore size) using Trans-Blot Turbo Transfer Packs (Bio-Rad) followed by blocking in TBS Intercept blocking buffer (LI-COR Biosciences, Lincoln, NE). Blots were incubated with the primary antibodies purchased from Cell Signaling Technologies (KIT clone Ab81, polyclonal phospho-KIT (Tyr823, #77522), p53 clone DO-7, β-Actin clone 13E5 or polyclonal #4967, GLI1 clone L42B10, GLI2 clone E7R1N) overnight or 1 h for β-Actin at 4°C on a shaker. Bands were detected using infrared-labeled secondary antibodies and imaging of the bands was performed using an Odyssey CLx Infrared Imaging System (LI-COR Biosciences). Quantification of infrared fluorescence was conducted using Image Studio Lite (version 5.2).

### Statistical Analysis

Statistical analysis was conducted using GraphPad Prism (GraphPad, San Diego, CA, version 9.1.1). The following significance scale was used on the graphs (* p<0.05, ** p<0.01, *** p<0.001, **** p<0.0001).

## Results

### Expression of HH Signaling Pathway Components and Modulation of GLI1/2 in Human Mast Cells

Activation of GLI proteins usually occurs downstream of the PTCH1/HH-pathway (**Figure 1A**), and which has not been explored in detail in mast cells. Thus, we first determined the level of mRNA expression of the main HH-pathway components in hMC and mast cell lines expressing normal (LAD2) or oncogenic KIT variants (HMC-1.1 and HMC-1.2). As shown in **Table 1**, PTCH1 mRNA was present in all human mast cell types but the key transducer of the HH-pathway, SMO, was not detectable in any cell type (Cq >39). The levels of the activator GLI1 were similar in all studied human mast cells, while the mRNA levels of GLI3 were barely detectable (Cq values of approximately 34). Notably, GLI2 mRNA was only expressed in the cell lines with KIT variants (HMC-1.1 and HMC-1.2 cells) and at higher levels than GLI1, suggesting a role for oncogenic KIT signaling in the regulation of GLI levels, particularly of GLI2. Related to such regulation, we found that inhibition of KIT, the downstream PI3K pathway, or particularly the MEK/ERK1/2 pathway, reduced GLI2 expression (**Supplementary Figure 1A**). The presence of GLI1 and GLI2 proteins, in accordance with the mRNA levels (**Table 1**), was confirmed by western-blot (**Supplementary Figure 1B**).

**Table 1:** Relative mRNA expression in human mast cells of the main genes involved in the Hedgehog signal transduction pathway.

	HMC-1.1		HMC-1.2		LAD2		hMC	
	*Mean ± SD*	*N*	*Mean ± SD*	*N*	*Mean ± SD*	*N*	*Mean ± SD*	*N*
**SHH**	ND	3	ND	3	ND	3	–	–
**PTCH1**	1.77 ± 0.40	3	1.78 ± 0.66	3	0.24 ± 0.010	2	3.02 ± 1.15	3
**GLI1**	0.49 ± 0.12	5	0.43 ± 0.28	6	0.37 ± 0.14	6	0.44 ± 0.19	5
**GLI2**	7.69 ± 0.23	4	15.72 ± 0.42	3	ND	3	ND	2
**GLI3**	0.020 ± 0.003	3	0.027 ± 0.012	3	0.027 ± 0.012	3	0.057 ± 0.021	3
**SUFU**	3.66 ± 0.48	2	4.68 ± 0.26	2	2.48 ± 0.96	3	6.73 ± 1.04	2
**SMO**	ND	3	ND	3	ND	3	ND	3

Mean values are normalized expression by GAPDH and ACTB (2^-ΔCq^). Mean and SD values are expressed as value x 10^3^. ND, Non-detected.

Since the levels of SMO mRNA (**Table 1**) and protein (not shown) were under the limit of detection, we questioned whether the canonical HH-pathway was functional in mature mast cells. As enhanced GLI1 expression is a main reporter assay for the activation of the HH-pathway (19, 28), we tested the effect of the PTCH1 agonist SHH or a pharmacological activator of SMO (SAG) (see **Figure 1A**) on GLI1 mRNA levels in hMC and mast cell lines. Neither of these approaches had significant effects on GLI1 mRNA levels after 6 h (not shown) or 20 h in hMC or mast cell lines (**Figure 1B**), consistent with the conclusion that the HH-pathway is not inducible in these cells under our experimental conditions.

SUFU is a key suppressor of GLI1 activity that can be regulated by SMO or by non-canonical pathways. Since SUFU was abundantly expressed in mast cells (**Table 1**), we used shRNA-mediated silencing of SUFU aiming to activate GLI bypassing upstream pathways. This approach resulted in 40 to 65% reduction in SUFU mRNA levels (**Supplementary Figure 2**) and led to an increase in GLI1 mRNA expression in all cell lines (**Figure 1C**), suggesting that the reduction of SUFU and thus SUFU/GLI complexes, allows GLI to enter the nucleus and further induce its own expression. The increased expression of GLI1 by silencing of SUFU implicates a role for SUFU in the regulation of GLI1 in mast cells and demonstrates that GLI1 is functionally active and capable of inducing its own expression, an outcome not seen by activation of PTCH1 or SMO (**Figure 1B**). This observation also suggests that SUFU/GLI may be mainly regulated *via* non-canonical pathways or that the induction of the canonical pathway is obscured by its homeostatic activation, although we did not detect SHH mRNA in these cells (**Table 1**).

Conversely to the knockdown of SUFU, the inhibition of GLI activation by a small molecule inhibitor, HPI-1, markedly reduced GLI1 and GLI2 mRNA expression in human mast cells, while no effect was observed with the SMO inhibitor vismodegib (**Figure 1D**). These data suggest that in cultured human mast cells, GLI transcription factors are active and regulated under steady state conditions by mechanisms other than the canonical HH-pathway.

### Homeostatic Activation of the HH-Pathway and Gli Activity Promotes Differentiation and Proliferation of Murine Mast Cells

**Figure 2:**
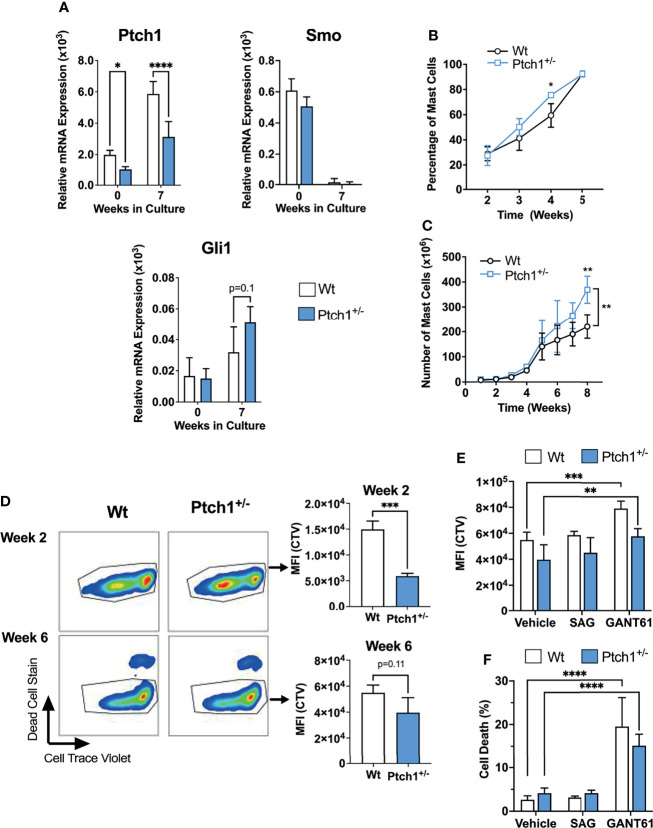
Ptch1 haploinsufficiency provides a proliferative advantage in mouse BMMC. **(A)** Relative mRNA levels (2^-ΔCq^) of Ptch1, Smo, and Gli1 in fresh bone marrow cells and after 7 weeks in culture, when >98% of cells are mast cells. Gapdh was used for normalization. **(B, C)** Percentage of mast cells (Kit^+^ FcεRI^+^ cells) **(B)** and total mast cell numbers **(C)** at the indicated times after initiation of the bone marrow culture (n=3 mice/group). Similar results were obtained in additional sets of cultures. The bracket in C indicates statistical significance between the two curves using 2-way ANOVA. The SD bars are not visible in some of the points due to the low variability between those cultures. **(D)** Proliferation assay using Cell Trace Violet (CTV) by FACS. BMMC were stained with the dye, at week 2 or 6 of culture. After washing, they were placed in culture media, and 6 days later, analyzed for CTV intensity and cell death staining. Representative dot-plots in FcϵRI^+^ Kit^+^ gated cells are shown on the left. On the right, the average of CTV median intensity in cultures is shown (n=3 mice/group). **(E, F)** CTV staining **(E)** and dead cell staining **(F)** of 6-week-old BMMC in the presence of the Smo agonist SAG (200 nM) or the Gli1/2 antagonist GANT61 (20 µM) for 6 days (n=3 mice/group). Results are shown as Mean ± SD. Unpaired t test or 2-way ANOVA followed by Dunnet multiple comparisons test were used for statistical analysis. *p < 0.05; **p < 0.01; ***p < 0.001; ****p < 0.0001.

To further investigate the potential consequences of homeostatic activation of the HH- pathway, we used a mouse model of haploinsufficiency of Ptch1. Ptch1 inhibits Smo in a catalytic manner, in that one molecule of Ptch1 can regulate about 50 molecules of Smo (29). Therefore, Ptch1 downregulation can result in significantly less Smo repression and in turn, enhanced activation of Gli1 (25, 30) (**Figure 1A**), representing some level of constitutive activation of the HH-pathway. We confirmed that Ptch1^+/-^ bone marrow cells and BMMC cultured for 7 weeks had significantly lower Ptch1 mRNA levels due to the lack of one allele (**Figure 2A**).

Although Ptch1^+/-^ BMMC developed into mast cells with similar appearance, expression of FcϵRI and Kit, and degranulation responses as Wt BMMC (**Supplementary Figure 3A, B**), we observed that mast cells developed slightly faster (note a significantly higher proportion of mast cells determined as FcϵRI^+^ Kit^+^ cells by 4 weeks; **Figure 2B**), and that the total number of Ptch1^+/-^ BMMC by the end of the culture was also significantly greater than in Wt BMMC (**Figure 2C**). We then tested the proliferation of mast cells during early development and in differentiated mast cells. By 2-3 weeks, when mast cells represent 20-30% of the cell culture, gated Ptch1^+/-^ BMMC showed, on average, a significant 3-fold reduction in CTV fluorescence intensity (due to dye dilution as cells proliferate) compared to gated Wt BMMC, while by 6 weeks, when mast cells represent >95% of the culture, the reduction in CTV fluorescence intensity in Ptch1^+/-^ cells was no more than 1.5 over Wt cells and did not reach statistical significance (**Figure 2D**). The trend towards reduced CTV in mature Ptch1^+/-^ BMMC cultures was however mirrored by a modest but statistically significant increase in cell numbers compared to Wt BMMC (**Supplementary Figure 3C**). This suggests that early in the development of mast cells, increased Ptch1/HH-pathway activity can promote mast cell differentiation and proliferation but the effect in proliferation diminishes in differentiated mast cells coinciding with a drop in the expression of Smo (**Figure 2A**), and reminiscent of the undetectable SMO expression in human mast cells (**Table 1**). In agreement with these data, the Smo agonist (SAG) had no effect on the proliferation of 6-week-old Wt or Ptch1^+/-^ BMMC (**Figure 2E**). In contrast, in the same 6-week-old cultures, inhibition of Gli1/2 by GANT61 reduced the proliferation of BMMC (**Figure 2E**) and caused a prominent increase in cell death (**Figure 2F**), potentially implicating Gli proteins in these processes.

Altogether, the data obtained from the haploinsufficiency Ptch1 mouse model system and inhibitors of the HH-pathway in BMMC are consistent with the conclusion that this pathway may contribute to the proliferation and differentiation of mast cells during early stages of development, but that the constitutive activation of the HH-pathway plays only a minor role in the proliferation of already differentiated mast cells. Of note, we did not detect differences in the numbers of tissue mast cells in these mice although the numbers trended to be slightly higher in Ptch1^+/-^ mice, suggesting that haploinsufficiency in the HH-pathway does not significantly affect mast cell homeostasis *in vivo* (**Supplementary Figure 3D**). Our findings also suggest that Gli activity, mostly conferred by non-canonical pathways and perhaps to a lesser extent through homeostatic Ptch1/Smo, may represent an important regulator of survival in differentiated mast cells.

### GLI1/2 Inhibition Reduces Human Mast Cell Growth by Downregulating Proliferation and Viability

Given these results and the regulation of GLI1 in human mast cells by the reduction in SUFU expression (**Figure 1C** and **Supplementary Figure 2**), we next focused on understanding the role of GLI proteins, particularly GLI1 and GLI2, in the regulation of human mast cell proliferation and survival. We employed the widely used GLI inhibitors GANT61 and ATO (31–33), and the new generation inhibitor HPI-1 (34), which as shown in **Figure 1D**, effectively downregulated both GLI1 and GLI2 mRNA expression, a readout of pathway inhibition.

**Figure 3:**
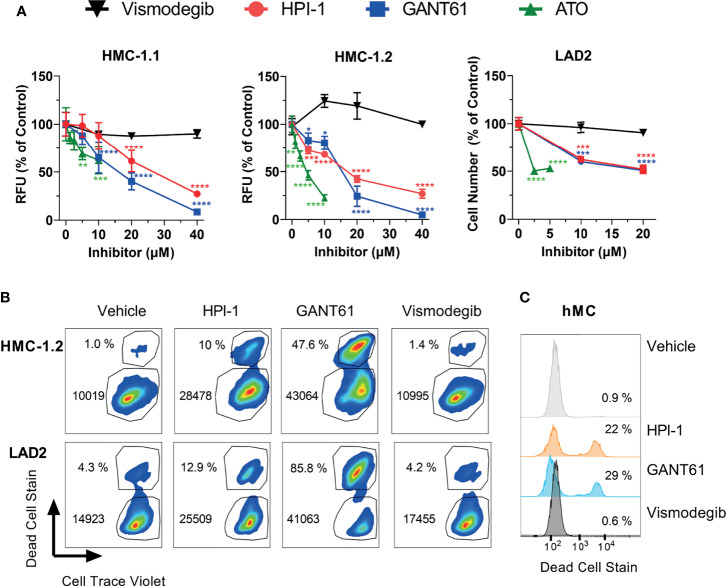
GLI1/2 inhibitors reduce human mast cell growth by decreasing viability and proliferation. **(A)** HMC-1.1 (left panel), HMC-1.2 (middle panel), and LAD2 (right panel) cells were cultured for 72 h (HMC-1.1 and HMC-1.2 cells) or 7 days (LAD2 cells) with increasing concentrations of the indicated inhibitors of GLI1/2 (HPI-1, GANT61 and ATO) or SMO (vismodegib). Cell growth was assessed by measuring relative units of fluorescence (RFU) that represent viable cells using a Cyquant assay; or counting viable cells with a cell counter (LAD2 cells). Results were normalized to vehicle control (represented as concentration 0 µM) and are expressed as Mean ± SD of three independent experiments. Two-way ANOVA followed by Dunnet multiple comparisons test (compared to vehicle) were used for statistical analysis. Approximated IC_50_ values, estimated using a non-linear fit between the inhibitor concentration and the normalized response (variable slope) with GraphPad Prism 9, were for HPI-1: 25 µM in HMC-1.1 and 16 µM in HMC1.2; and for GANT61: 15 µM in HMC-1.1 and 14 µM in HMC1.2**. (B)** Representative dot-plots of three independent experiments in which HMC-1.2 and LAD2 were cultured for 72 h or 7 days, respectively, in the presence of the indicated inhibitors (20 µM). Cells were stained with Cell Trace Violet (CTV) before the incubation and at the end of the experiment stained with green dead cell stain. The percentage of dead cells and median fluorescence intensity (MFI) for CTV is shown in each dot-plot. **(C)** Representative histograms of primary human mast cells (hMC; 1 healthy donor out of 2) cultured for 5 days with the indicated inhibitors (20 µM) and stained with green dead cell stain. Averages of CTV MFI and percentages of dead cells in three independent experiments are shown in **Supplementary Figure 4**. *p < 0.05; **p < 0.01; ***p < 0.001; ****p < 0.0001.

All GLI inhibitors markedly reduced (by 40 to 95%) the growth of neoplastic human mast cells in culture (**Figure 3A**) in a concentration-dependent manner (**Figure 3A**). We used concentrations ranging from 5 to 40 µM of GANT61 and HPI-1, or up to 10 µM of ATO, as these were within the range of the reported IC_50_ for the corresponding inhibitors in several cell line models (31, 34–36). Concentrations greater than 40 µM for HPI-1 and GANT61, or greater than10 µM for ATO were not tested due to concerns of potential off-target effects. The reductions in cell growth were observed at 72 h in HMC-1.1 and HMC-1.2 cells (**Figure 3A**, left and middle panels), while in the slower growing LAD2 cells, a reduction of about 50% in cell number was observed after 7 days in culture (**Figure 3A**, right panel). In contrast, vismodegib, an FDA-approved antagonist of SMO, did not show a significant effect in either GLI1 mRNA expression (**Figure 1D**) or cell growth of either cell line (**Figure 3A**).

Using flow cytometry assays on LAD2 and HMC-1.2 cells as examples of neoplastic cells without or with oncogenic KIT variants, we were able to demonstrate that GLI inhibition by 20 µM GANT61 or HPI-1, a concentration near the approximated IC50 (**Figure 3A**), not only reduced cell proliferation (indicated by the higher median CTV fluorescence intensity), but also caused a prominent 3-to-26-fold increase in cell death (**Figure 3B** and **Supplementary Figure 4**), similarly to our results in BMMC (**Figures 2E, F**). In contrast, vismodegib did not show any noticeable effects on cell proliferation or cell death (**Figure 3B**). Although primary human mast cells do not proliferate after 5 weeks in culture (and thus proliferation could not be tested), their survival was also significantly reduced after GLI inhibition but not after SMO inhibition (**Figure 3C** and **Supplementary Figure 4**). Altogether, these results suggest that in normal or neoplastic mast cells, GLI transcription factors play a key homeostatic role in the proliferation and/or survival of mast cells largely *via* non-canonical pathways.

**Figure 4:**
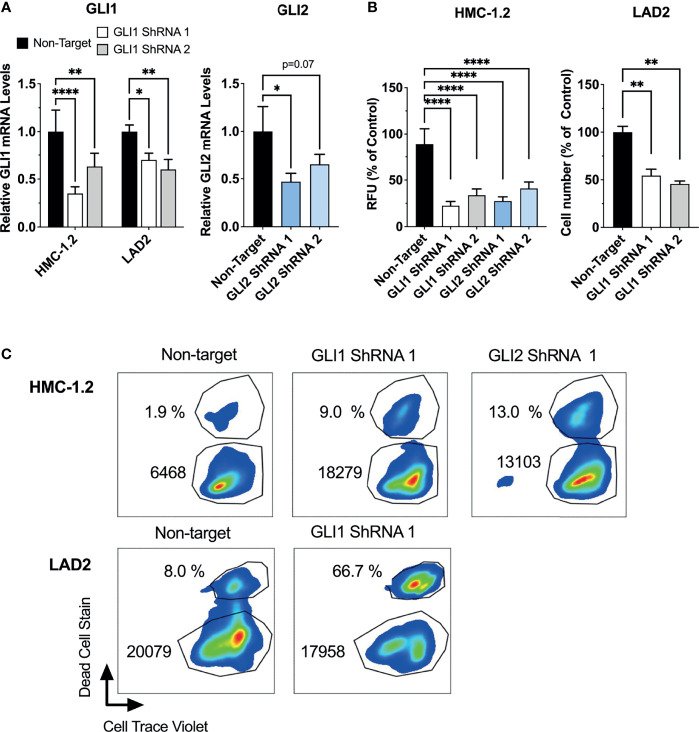
ShRNA-mediated silencing of GLI1/2 reduces human mast cell growth by decreasing viability and proliferation. **(A)** HMC-1.2 and LAD2 cells were transduced with lentiviral particles containing GLI1 or GLI2 shRNA constructs (two independent constructs for each). After proper selection, relative GLI1 and GLI2 mRNA levels (2^-ΔΔCq^) were quantified by qPCR. GAPDH and ACTB were used for normalization. **(B)** After proper selection numbers of viable HMC-1.2 (left) and LAD2 (right) cells were assessed using the Cyquant assay (relative fluorescence units, RFU) or a cell counter, respectively. Cell numbers were assessed after 72 h in HMC-1.2 cells and after 7 days in LAD2 cells. Results are expressed as Mean ± SD of three independent experiments. One-way or 2-way ANOVA followed by Dunnet multiple comparisons test were used for statistical analysis. **(C)** After proper selection, cell proliferation and cell death were assessed as described in **Figure 3**. Representative dot-plots of two independent experiments are shown. The percentage of dead cells and median fluorescence intensity (MFI) of Cell Trace Violet (CTV) fluorescence within the live cells gate are shown in each dot-plot. Averages of CTV MFI and percentages of dead cells in two independent experiments are shown in **Supplementary Figure 5**. *p < 0.05; **p < 0.01; ****p < 0.0001.

To demonstrate that growth inhibition was not due to off-target effects of these drugs, we also performed GLI1 or GLI2 specific shRNA-mediated silencing in HMC-1.2 and LAD2 cells, using at least two independent constructs for each. We did not target GLI2 in LAD2 cells since GLI2 expression was not detected in these cells (**Table 1**). It is worth noting that a correlation between the reduction in GLI mRNA and the functional effects may not be linear because cells with a more efficient reduction in GLI1 or GLI2 are more likely to die during the course of the experiments (when knockdown efficiency was assessed), while those cells where the knockdown was less efficient may remain alive. Nevertheless, effective GLI1 or GLI2 silencing (by about 50%; **Figure 4A**) reduced the growth of HMC-1.2 cells by more than 50% (**Figure 4B**, left panel) in 72 h. Similarly, knockdown of GLI1 in LAD2 cells (**Figure 4A**) reduced cell numbers by 50% in 7 days (**Figure 4B**, right panel). Using flow cytometry assays, GLI silencing reduced both the proliferation and survival of HMC-1.2 as well as the survival of LAD2 cells (**Figure 4C** and **Supplementary Figure 5**), confirming a specific role for GLI1/2 in these biological processes.

### GLI1/2 Inhibitors Induce Apoptosis and Regulate p53 in Human Mast Cells

**Figure 5:**
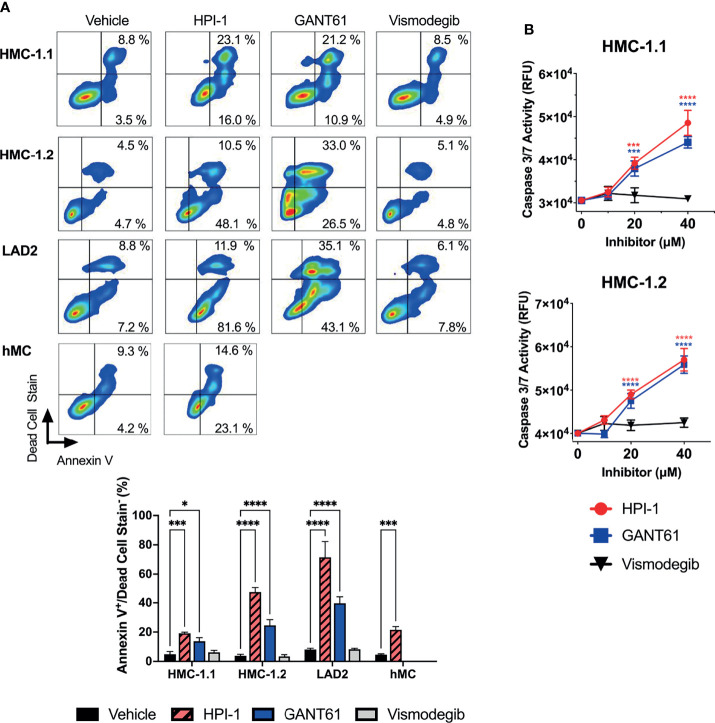
GLI1/2 inhibition causes apoptosis in human mast cells. **(A)** HMC-1.1, HMC-1.2, LAD2, and primary human mast cells (hMC) were cultured in the presence of HPI-1, GANT61, or vismodegib (20 µM) for 48 h (HMC-1.1 and HMC-1.2 cells), 6 days (LAD2 cells) and 4 days (hMC). The percentages of annexin V positive and dead cells were quantified by flow cytometry. Representative dot-plots from one of three independent experiments are shown. The bar graph represents the average percentage of apoptotic cells (annexinV^+^/dead cell stain^-^) in three independent experiments using the indicated cells and treatments. **(B)** Caspase 3/7 activity was quantified by measuring relative units of fluorescence (RFU) of a caspase 3/7 substrate in HMC-1.1 and HMC 1.2 after 48h incubation with increasing concentrations of inhibitors, vehicle control is represented as concentration 0 µM. Results expressed as Mean ± SD of three independent experiments. Two-way ANOVA followed by Dunnet multiple comparisons test (compared to vehicle) were used for statistical analysis. *p < 0.05; ***p < 0.001; ****p < 0.0001.

We next used annexin V staining and flow cytometry to assess the type of death induced by the loss of GLI activity. The increase in annexin V staining after GLI1/2 inhibition indicated cell death by apoptosis (**Figure 5A**), a finding that was confirmed by directly measuring caspase 3/7 activity in cultures 48 h after treatment with GLI1/2 inhibitors (**Figure 5B**). Unlike HPI-1 and GANT61, vismodegib did not affect either annexin V staining (**Figure 5A**) or the activity of caspase 3/7 (**Figure 5B**), underlining a function for GLI proteins in mast cell survival that is largely independent of SMO regulation.

**Figure 6:**
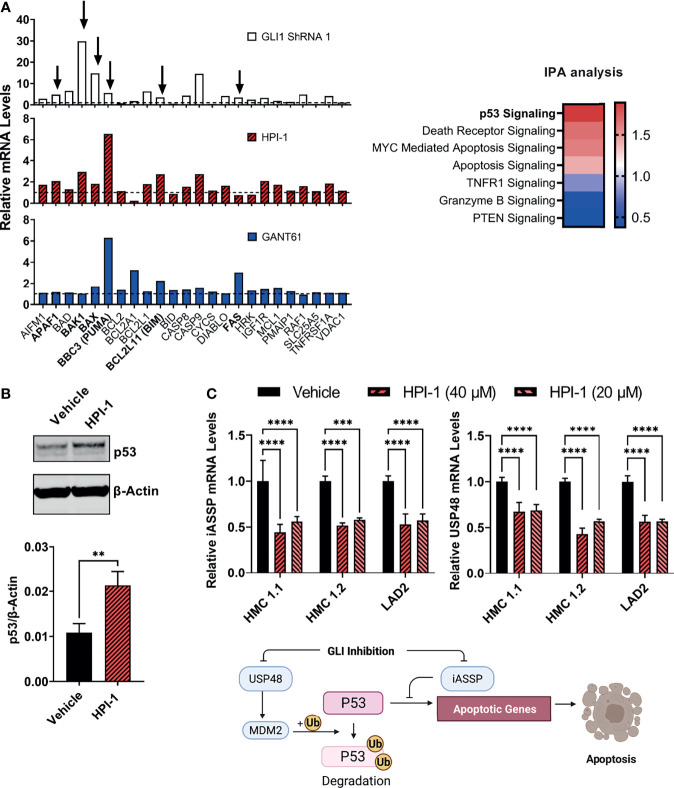
GLI1 inhibition stabilizes p53 and alters the gene expression of pro-apoptotic proteins. **(A)** Gene expression profiling of differentially expressed genes (2^-ΔΔCq^) within the mitochondrial apoptotic pathway (p53-regulated genes are marked by arrows and in bold). RNA from HMC-1.2 cells was extracted after an overnight incubation with HPI-1 or GANT61 (40 µM) or after proper selection of GLI1 shRNA (construct 1)-transduced cells. Vehicle and non-target mRNA levels are represented by dotted lines. Z-scores heatmap of the top seven canonical pathways modulated in GLI1 ShRNA (construct 1)-transduced cells, determined by using an IPA analysis. **(B)** HMC-1.2 cells were incubated overnight in the presence of HPI-1 (40 µM), cell lysates were obtained and p53 was quantified by western blot. β-actin was used as a loading control. The western blot shows a representative experiment and the average quantification of p53 protein expression, normalized to β-actin, in 3 independent experiments, are shown in the bar graph (Mean ± SD). Full-length blots are shown in **Supplementary Figure 6A**. **(C)** RNA from human mast cell lines was obtained after incubation for 5 h with HPI-1 (20 or 40 µM) and relative mRNA levels (2^-ΔΔCq^) of iASSP and USP48 was determined by qPCR. GAPDH and ACTB were used for normalization. Results are expressed as Mean ± SD of three independent experiments. Illustration represents the regulation of p53 levels and function by iASSP and USP48. Unpaired t test or 2-way ANOVA followed by Dunnet multiple comparisons test were used for statistical analysis. **p < 0.01; ***p < 0.001; ****p < 0.0001.

In addition, we performed gene expression profile arrays of apoptotic pathways in the aggressive neoplastic HMC-1.2 mast cell line. The arrays showed that inhibition of GLI activity by HPI-1 and GANT or by GLI1 silencing *via* shRNA caused an upregulation of genes involved in the mitochondrial or intrinsic apoptotic pathway. Ingenuity Pathway Analysis of the genes upregulated in these expression arrays indicated activation of p53 as one potential upstream regulator of the apoptotic process (**Figure 6A**, right). Indeed, BBC3 (PUMA), BCL2L11 (BIM), and BAX, and to some extent APAF1, FAS and BAK1, all reported transcriptional targets of p53 (37, 38), were generally increased with all or most of the treatments with GANT61, HPI-1 or GLI1-ShRNA construct (**Figure 6A**), suggesting an upregulation of p53 function by loss of GLI activity.

An increase in p53 protein levels after inhibition of GLI was then corroborated by immunoblotting (**Figure 6B**). GLI1/2 proteins have been reported in other cells to induce the transcription of the deubiquitinase USP48 (39), which can bind and stabilize Mdm2 leading to the degradation of p53 (40), and of iASPP, an inhibitor of p53-induced transcription (41). Next, we analyzed the gene expression of these antiapoptotic proteins after GLI1/2 inhibition. Treatment of HMC-1.1, HMC-1.2 or LAD2 cells with HPI-1 caused a 50% reduction in the mRNA levels of USP48 and iASPP (**Figure 6C**), suggesting that homeostatic GLI activity regulates p53 stability and transcriptional activity by regulating respectively USP48 and iASSP, and that loss of GLI activity causes an increase in p53-mediated apoptotic cell death, thus linking transcriptional regulatory function of GLI to the control of mast cell apoptosis (see illustration in **Figure 6C**).

### GLI1 Inhibition Downregulates KIT Expression in Human Mast Cells

**Figure 7:**
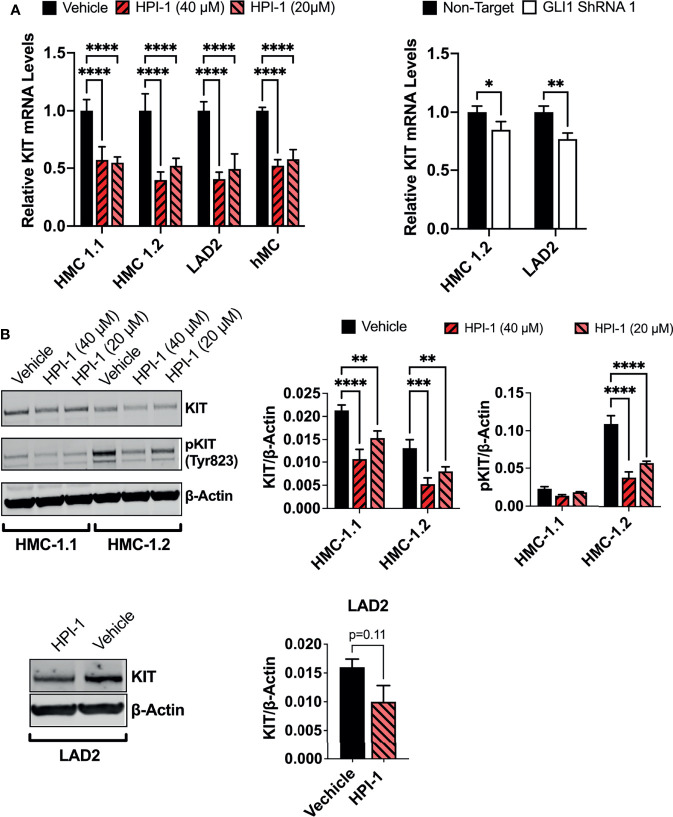
GLI1 inhibition downregulates KIT expression and phosphorylation in human mast cells. **(A)** Relative KIT mRNA levels (2^-ΔΔCq^) in mast cell lines or primary human mast cells (hMC) after 5h incubation with HPI-1 (20 or 40 µM) or 48h after transduction with lentiviral particles containing GLI1 shRNA (construct 1), no puromycin selection was conducted. Results are expressed as Mean ± SD of three independent experiments. GAPDH and ACTB were used for normalization. **(B)** HMC-1.1, HMC-1.2 and LAD2 cells were cultured overnight with HPI-1 (20 or 40µM) and lysed. Proteins were resolved in SDS-PAGE. KIT and phosphorylated-KIT (pKIT) were quantified by western-blot. β-actin was used as a loading control. The blots show representative images, and the bar graphs are the average relative band intensities of KIT and phospho-KIT (normalized by β-actin) in 2 independent experiments (Mean ± SD). Unpaired t test or 2-way ANOVA followed by Dunnet multiple comparisons test were used for statistical analysis. *p < 0.05; **p < 0.01; ***p < 0.001; ****p < 0.0001. Full-length blots are shown in **Supplementary Figure 6B**.

The HH-pathway and GLI3 have been implicated in the regulation of KIT expression (14). In agreement, inhibition of GLI1 *via* HPI-1 or GLI1 shRNA reduced KIT mRNA in the human mast cell lines and primary mast cell cultures (**Figure 7A**), with accompanying reductions in KIT protein levels (**Figure 7B**). It is important to note that the reduction in KIT occurred regardless of the presence of oncogenic KIT variants in mast cells, and the constitutive phosphorylation of KIT in HMC-1.2 cells, harboring V560G and D816V, was also prominently reduced (**Figure 7B**), making targeting of GLI1/2 another potential avenue for controlling mast cell numbers even in dysplastic diseases with aggressive KIT mutations.

### Gli1/2 Inhibitors Reduce Mast Cell Numbers *In Vivo*

**Figure 8:**
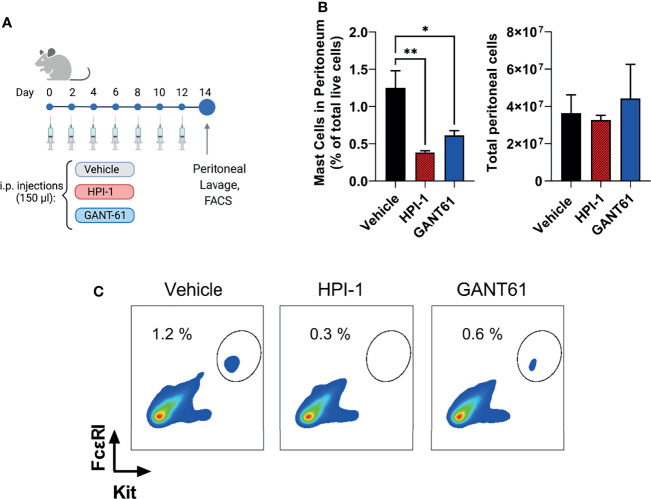
Gli1/2 inhibitors reduce peritoneal mast cell numbers *in vivo*. **(A)** Scheme illustrating the protocol for treatment of mice with the indicated Gli1/2 inhibitors. **(B)** Peritoneal lavage was obtained 2 days after the last injection with vehicle control (PBS : EtOH; 95:5%), HPI-1 (2 mg/Kg), or GANT61 (2 mg/Kg). Cells were stained with anti-FcϵRI and anti-Kit antibodies and separated by FACS. Percentages of mast cell (FcϵRI^+^ Kit^+^ cells) among total live peritoneal cells for each treatment are shown (left). The number of viable cells in the peritoneal lavage after these treatments was measured using a cell counter (right panel). Results are expressed as Mean ± SD (n=4 mice/group). One-way ANOVA followed by Dunnet multiple comparisons test were used for statistical analysis. **(C)** Representative dot-plots from one mouse per group are shown. *p < 0.05; **p < 0.01.

To demonstrate that the effects of Gli1/2 inhibition were not restricted to mast cells in culture, we determined the numbers of peritoneal mast cells in mice after successive injections with HPI-1 or GANT61 (**Figure 8A**). These inhibitors, used at concentrations 10 to 37 times lower (2 mg/Kg) than those used in xenograph *in vivo* models (20-75 mg/kg) (42–44), significantly reduced the number of peritoneal mast cells identified as FcϵRI^+^/Kit^+^ cells (**Figures 8B** left, and **8C**). In contrast, the total number of peritoneal cells were not significantly changed (**Figure 8B** right). These results provide proof of principle that GLI proteins have a function in the survival of mast cells in tissue and offer GLI pathway components as potential targets for mast cell depletion in tissues. Further characterization of the effects on specific cell populations is warranted when considering the use of these drugs for specific depletion of mast cells *in vivo*.

## Discussion

Mast cells are effector cells in allergic inflammation and play important roles in immune surveillance. In normal tissue, the numbers of mast cells are maintained constant but can increase significantly in disease conditions and contribute to mast cell-related symptoms. Although mast cell replenishment and expansion can occur by differentiation from mast cell precursors in tissue (45–47), their numbers are also determined by signals from growth factor and cytokine receptors that regulate mature mast cell proliferation and death (3). The understanding of these fundamental processes and the intracellular regulatory components of mast cell homeostasis remain incomplete (2, 3). In this study, we demonstrate that GLI1/2 family members are critical transcription factors that reduce apoptotic signals and heighten proliferation of human and mouse mast cells in culture and regulate mast cell homeostasis *in vivo*. Our findings also provide insight into the underlying mechanisms for GLI actions and offer targets for potential development of therapies to control dysregulated mast cells.

Among the transcription factors known to regulate mast cell numbers *in vitro* and *in vivo*, GATA2 and MITF are notable in mast cell lineage specification and function (48–50), while STAT5 is known to regulate proliferation and survival of mast cells (3, 51). Here, we implicate the transcription factors GLI1 and GLI2 as additional key players in the steady-state regulation of the proliferation and survival of normal and transformed mast cells. This conclusion is based on the findings that inhibition of GLI1/2 by small molecules or by single shRNA-mediated knockdown effectively and markedly reduced the growth of human mast cell lines, particularly the aggressive, highly proliferative HMC-1.1 and HMC-1.2 cell lines and caused a marked drop in mast cell viability that was common to primary and neoplastic human mast cells. Furthermore, Gli1/2 inhibitors, when injected peritoneally into mice, also significantly diminished peritoneal mast cell numbers, indicating a function for Gli in the survival of mouse mast cells *in situ*.

The effect of GLI1/2 suppression on reduced human mast cell viability was accompanied by an increase in apoptotic markers. Analysis of gene expression arrays followed by western-blot assays, led us to associate the apoptotic process with an increase in the levels and activity of the tumor suppressor p53. Some reports have implicated p53 in GLI-mediated regulation of proliferation and apoptosis (41, 52–54), while in others, GLI1/2 inhibition induced p53-independent apoptosis (55, 56). In human mast cells, the increase in p53 activity induced by GLI inhibition was associated with a downregulation of two antiapoptotic proteins known to impact p53 stability and function: USP48 and iASSP, respectively (see illustration in **Figure 6C**). USP48 binds MDM2 to promote MDM2-mediated p53 ubiquitination and degradation (40), while iASPP is a p53 cofactor that inhibits p53 ability to transactivate pro-apoptotic target genes (57). In support for a cause-effect relationship between GLI inhibition and the reduction in USP48 and iASSP mRNA levels, USP48 was found to be a direct transcriptional target of GLI1 in glioblastoma cells (39), and iASPP to be induced by GLI1/2 activation *via* E2F1 in melanoma cells (39, 41). Cytokines that suppress mast cell growth and survival such as TGF-β, IL-4 and IL-10 (58) and drugs that induce mast cell apoptosis (59, 60) often require p53 activation and expression of apoptotic proteins (PUMA, APAF, BIM and BAX) that counteract the antiapoptotic BCL2 family members (Bcl2, Bclxl and MCL1), which are known to extend mast cell survival (13, 61–63). This underscores the importance of p53 regulation in mast cell biology. Our data is consistent with the conclusion that GLI proteins, in standard cell culture media conditions, and in the absence of additional exogenous stimuli, act as negative modulators of p53 by controlling the expression of USP48 and iASPP, which offers mechanistic insights for p53 regulation in mast cells as well as potential targets to induce mast cell depletion.

Thus, the steady-state action of GLI acts as a gatekeeper to repress p53, therefore favoring mast cell survival. The activity of GLI proteins is usually studied in the context of the canonical HH signaling, although GLI can also be activated by non-canonical pathways (17, 19, 64, 65). Our data overall does not advocate a major role for a canonical activation of GLI. Autocrine pathway induction *via* constitutive production of HH ligands is unlikely since we did not find mRNA for the most broadly expressed HH ligand, SHH, in any of the human mast cells. The canonical HH-pathway involves activation of PTCH1 which allows for the required translocation of SMO to the primary cilium and consequent GLI activation (66). The primary cilium is a sensory organelle considered to be present in most vertebrate cells (67) except for lymphoid and myeloid cells (68). In support, hematopoietic-specific deletion of Ptch1 did not lead to activation of the HH signaling pathway (69). In another report, however, primary cilia were documented to be present in most human blood and bone marrow cells, although their lineage was not determined (70). It is currently unknown whether mast cells, of myeloid origin, have a primary cilium to support this pathway. Nonetheless, our results altogether contradict the presence of a functional canonical HH-pathway in these cells: 1) SMO expression was not detectable in differentiated mast cells; 2) activation of the pathway by a PTCH1 ligand or a SMO agonist (SAG) did not result in GLI expression or affected human mast cell proliferation and survival; and 3) GLI expression and these biological functions were not altered by inhibition of SMO with the antagonist vismodegib.

This conclusion differs from a recent report that describes a haploinsufficient heterozygous mutation in GLI3 in a subpopulation of congenital mastocytosis with Greig cephalopolysyndactyly syndrome and implicates the canonical HH-pathway in aggressive mastocytosis and cultured neoplastic mast cells (16). In contrast to our work, this study found that SMO antagonists, particularly sonidegib, reduced the growth of neoplastic HMC-1.1 and HMC-1.2 cells. These effects were seen in longer times than the 3 days we used in our experiments, when cells have further expanded. A possible explanation for this discrepancy might be the confluency of the cells in the two studies, since other reports have shown that cultured cells only express cilia and become HH responsive when they are confluent (71, 72). In addition, it is important to keep in mind potential off target effects of these drugs after prolonged treatments. Nevertheless, our study agrees with that of Polivka et al. (16) in that the activation of GLI in human mast cells includes a prominent non-canonical component resulting in mast cell growth/survival. Further studies are needed to determine if the canonical pathway can be induced *in vivo* or under particular experimental conditions, and how exactly GLI activity is maintained and regulated in human mast cells by non-canonical pathways.

Related to the canonical pathway, our experiments using a mouse model of Ptch1 haploinsufficiency that can achieve some level of constitutive HH-pathway activation, showed that a reduction in Ptch1 expression provides a proliferative advantage to BMMC before the culture reaches full maturity. However, the functional effect of Ptch1 haploinsufficiency was minimal in differentiated BMMC, consistent with our data in human mast cells that argues against a role for the canonical HH-pathway. Since early cultures contain mixed cell lineages, these experiments cannot discern whether the HH-pathway is intrinsically important for differentiation and proliferation of mouse mast cell progenitors, or it is secondary to the activation of this pathway on other cell types. This later view is supported by a report where only deletion of Ptch1 in non-hematopoietic cells, but not deletion of Ptch1 in hematopoietic cells, caused dramatic hematopoietic phenotypes including increased circulating myeloid progenitors (69). Polivka et al. (16) also showed the presence of SHH in biopsies of the skin, digestive tract, and bone marrow of patients with mastocytosis, environments where mast cells were present and thus the HH-pathway could also affect mast cells or mast cell progenitors *via* mast cell-extrinsic mechanisms.

Many transcription factors that regulate mast cell homeostasis, including MITF, GATA2 and STAT5 influence the expression of KIT. Conversely, KIT regulate the expression/activation status of these transcription factors (3, 48–51, 73). KIT is a central receptor providing positive signals for mast cell survival and proliferation. Previous reports have shown or suggested that GLI transcription factors regulate KIT expression (14, 16). We provide confirmation that GLI1/2 inhibition suppressed KIT expression at the mRNA and protein levels in human mast cells, regardless of oncogenic mutations, an effect that may also represent a cause for the drop in viability and proliferation in the cells. Reciprocally to GLI regulating KIT expression, KIT signals were described to regulate GLI expression (16). Although we did not explore this possibility, our results showed that the expression of GLI2 (only detected in HMC-1.1 and HMC-1.2 cells) is regulated by KIT oncogenic signals, mainly the MEK/ERK1/2 pathway and to some extent the PI3K pathway (**Supplementary Figure 1A**) suggesting positive feedback loops between KIT and GLI. The increase in GLI2 expression in the rapidly growing neoplastic HMC-1.1 and HMC-1.2 cells (**Table 1** and **Supplementary Figure 1**) is consistent with findings that increased expression of GLI^A^ can lead to transformation (18, 19).

In summary, the data presented here support a role for GLI transcription factors as molecular rheostats of mast cell preservation. Homeostatic GLI function, modulated in cultures mainly by non-canonical pathways, controls p53 function and KIT expression, resulting in suppression of apoptosis and promoting proliferation of mast cells, thus contributing to mast cell number maintenance in steady-state and possibly in disease conditions with mast cell-related presentations, as shown in mastocytosis (16). Future studies are warranted to gain a better understanding of GLI regulation in mast cells in their local environment and to develop targeted intracellular delivery mechanisms for inhibitors of GLI and its targets to control mast cell numbers when needed.

## Data Availability Statement

The original contributions presented in the study are included in the article/**Supplementary Material**. Further inquiries can be directed to the corresponding author.

## Ethics Statement

The studies involving human participants were reviewed and approved by National Institutes of Health Internal Review Board, protocol# NCT00001756. The patients/participants provided their written informed consent to participate in this study. The animal study (under protocol LAD2E) was reviewed and approved by NIAID-DIR-Animal Care and Use Committee.

## Author Contributions

GF designed and conducted experiments, analyzed data, prepared figures, and drafted the manuscript. AP, QZ, and YY performed experiments and analyzed data. AO supervised the study, designed and helped with experiments, prepared figures, and wrote the manuscript. DM supervised the study, wrote the manuscript, and provided funding. All authors approved the manuscript.

## Funding

This work was supported by the Division of Intramural Research of the National Institutes of Health (NIH), National Institute of Allergy and Infectious Diseases (NIAID).

## Conflict of Interest

The authors declare that the research was conducted in the absence of any commercial or financial relationships that could be construed as a potential conflict of interest.

## Publisher’s Note

All claims expressed in this article are solely those of the authors and do not necessarily represent those of their affiliated organizations, or those of the publisher, the editors and the reviewers. Any product that may be evaluated in this article, or claim that may be made by its manufacturer, is not guaranteed or endorsed by the publisher.

## Abbreviations

GLI, Glioma-associated oncogene family; HH, hedgehog pathway; SHH, sonic HH ligand; SAG, SMO agonist; GLI^A^, GLI activator forms; GLI^R^, GLI repressor forms; ATO, arsenic trioxide/A_2_O_3_; BMMC, bone marrow-derived mast cells; hMC, primary human mast cells.
