# Supplementary material for: A Critical Function for the Transcription Factors GLI1 and GLI2 in the Proliferation and Survival of Human Mast Cells
Source: Front Immunol. 2022 Feb 16;13:841045. doi: 10.3389/fimmu.2022.841045 (PMC8888842; doi:10.3389/fimmu.2022.841045)
Supplement: Supplementary file 1 [file DataSheet_1.pdf]

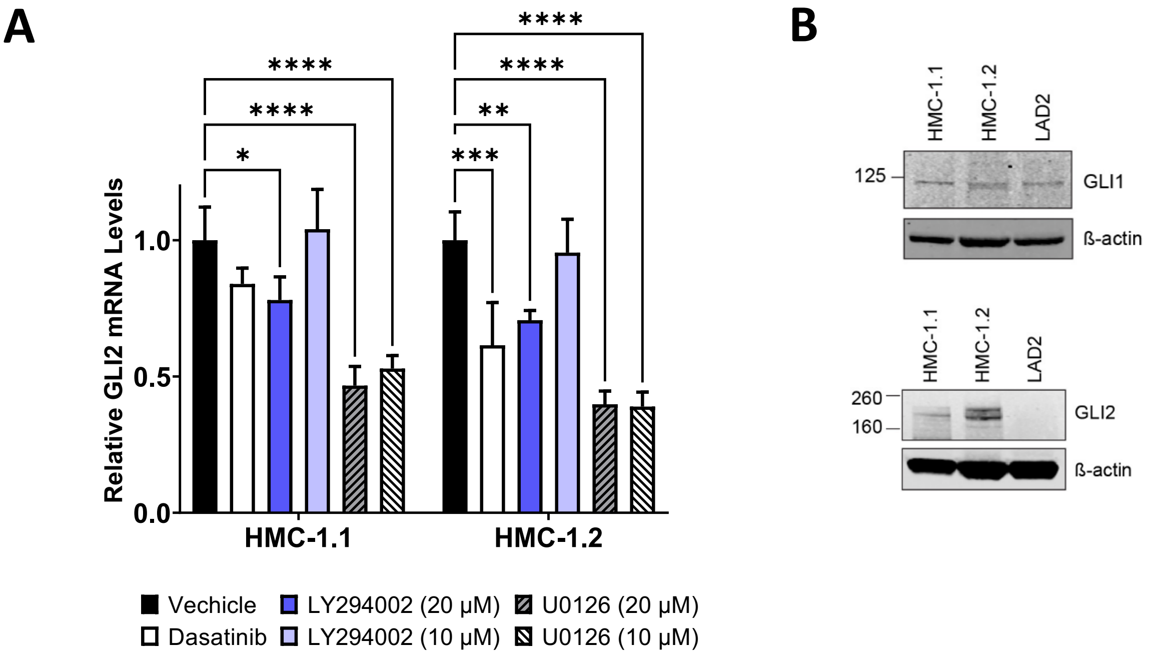

Supplementary Figure 1. (A) Modulation of GLI2 mRNA expression by oncogenic KIT signaling in HMC-1 cells. Relative GLI2 mRNA levels ($2^{-\Delta\Delta Cq}$) after 5h incubation with the TKI dasatinib (0.5 µM), the PI3Kα/δ/β inhibitor LY294002 (20 or 10 µM), or the MEK1/2 inhibitor U0126 (20 or 10 µM). GAPDH and ACTB were used for normalization. Results are expressed as Mean±SD of three independent experiments. Each individual experiment was done in triplicate. Two-way ANOVA followed by Dunnet multiple comparisons test was used for statistical analysis. **(B) GLI1 and GLI2 protein expression in human mast cell lines.** Proteins from HMC-1.1, HMC-1.2, and LAD2 lysates were resolved in SDS-PAGE and GLI1 and GLI2 expression was assessed by western-blot. β-actin was used as a loading control.

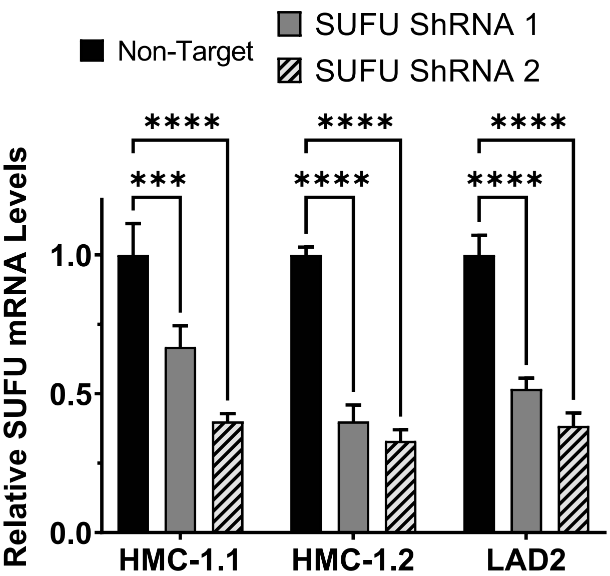

Supplementary Figure 2. Relative SUFU mRNA levels ($2^{-\Delta\Delta Cq}$) in mast cell lines transduced with lentiviral particles containing two separate ShRNA constructs to knockdown SUFU (See Figure 1D experiment). GAPDH and ACTB were used for normalization. Results are expressed as Mean±SD of three independent experiments. Each individual experiment was done in triplicate. Two-way ANOVA followed by Dunnet multiple comparisons test was used for statistical analysis.

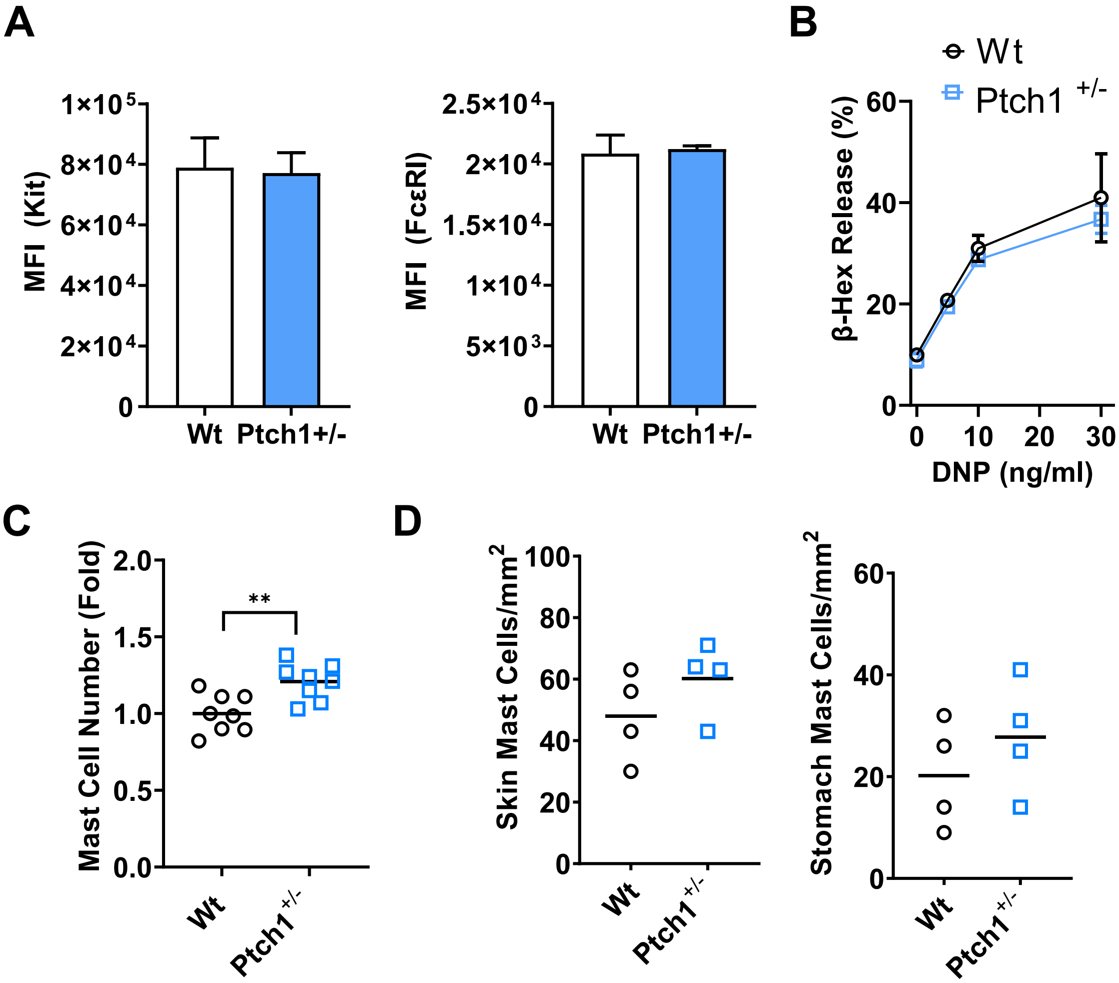

Supplementary Figure 3. (A) Average of Kit and FcεRI Median Fluorescence intensity (MFI) in fully matured BMMC cultures (3 mice/group). **(B)** Analysis of degranulation in BMMC. Fully matured mast cells (7-weeks-old; 3 mice/group) were sensitized with 100 ng/mL anti-dinitrophenyl (DNP)-IgE in cytokine-free media for 14 h. Degranulation was assessed by measuring the release of β-hexosaminidase into the media after 30 min of stimulation with the indicated concentrations of DNP. Results are expressed as Mean±SD. **(C)** Changes in numbers of Wt or Ptch1$^{+/-}$ BMMC (6-8 weeks old) 7 days after seeding in full media containing IL-3. Each dot represents a BMMC culture from a different mouse. Experiments were conducted 3 separate times (sets), each containing 2-3 individual cultures/genotype. Wt cell numbers for each set of experiments were averaged and used to calculate individual fold changes. **(D)** Average number of

mast cells per mm^2 of skin or stomach tissue samples from adult Wt or Ptch+/- mice (4 mice/group). Tissues were fixed in buffered paraformaldehyde, paraffin-embedded and stained with toluidine blue with eosine for contrast. Slides were scanned using a NanoZoomer S60 (Hamamatsu) and dark purple stained mast cells were counted. Unpaired Student t-tests were used for statistical analysis.

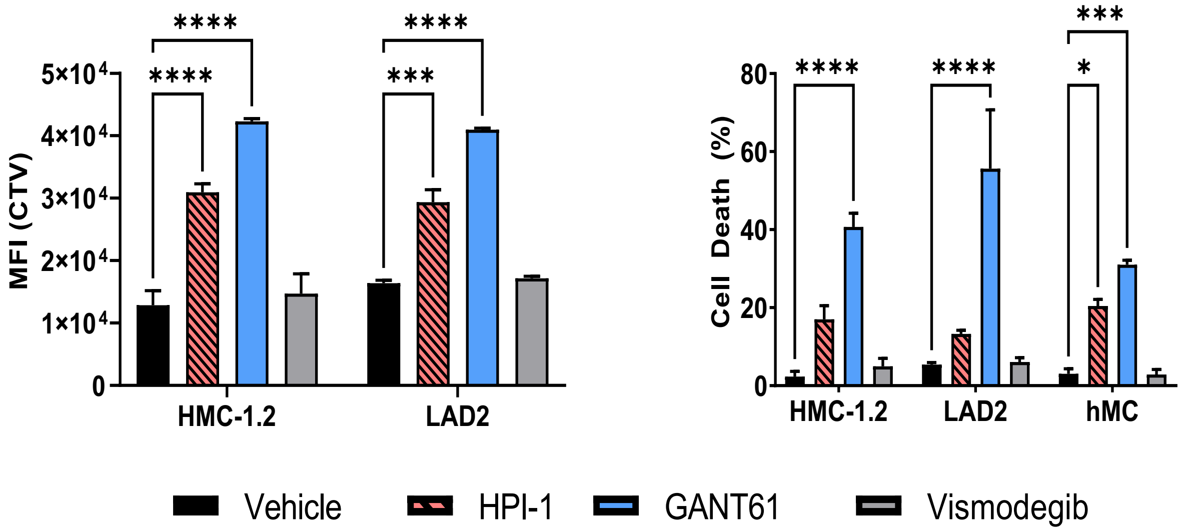

Supplementary Figure 4. GLI1/GLI2 inhibitors reduce human mast cell viability and proliferation. Data analysis from Figure 3B and C assays. HMC-1.2 and LAD2 were cultured for 72 h or 7 days, respectively, in the presence of the indicated inhibitors (20 µM). Cells were stained with Cell Trace Violet (CTV) before the treatments and at the end of the experiment they were stained with green dead cell stain. Primary hMC (2 healthy donors) were cultured for 5 days with the indicated inhibitors (20 µM) and stained with green dead cell stain. The means of the median fluorescence intensity (MFI) of CTV fluorescence within the live cells gate (left panel) and the percentage of dead cells (right panel) are shown. Results are expressed as Mean±SD of three independent experiments. Two-way ANOVA followed by Dunnet multiple comparisons test was used for statistical analysis.

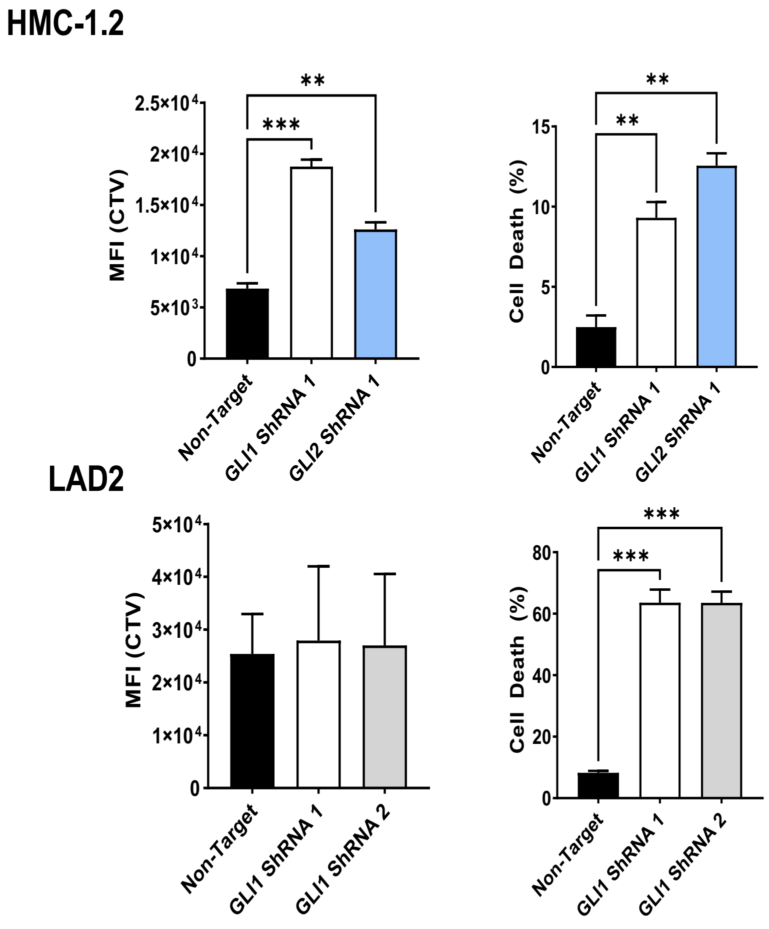

Supplementary Figure 5. ShRNA-mediated silencing of GLI1/GLI2 reduces human mast cell viability and proliferation. Data analysis from Figure 4C assays, showing the average of two independent experiments. After proper selection, cell proliferation (left panels) and cell death (right panels) were assessed as described in supplementary Fig. 4. Note that for LAD2 cells we used two constructs to knockdown GLI1 (GLI1 ShRNA 1 and GLI1 ShRNA 2), but no constructs for GLI2 were used since LAD2 did not express any GLI2. Results are expressed as Mean±SD of two independent experiments. One-way ANOVA followed by Dunnet multiple comparisons test was used for statistical analysis.

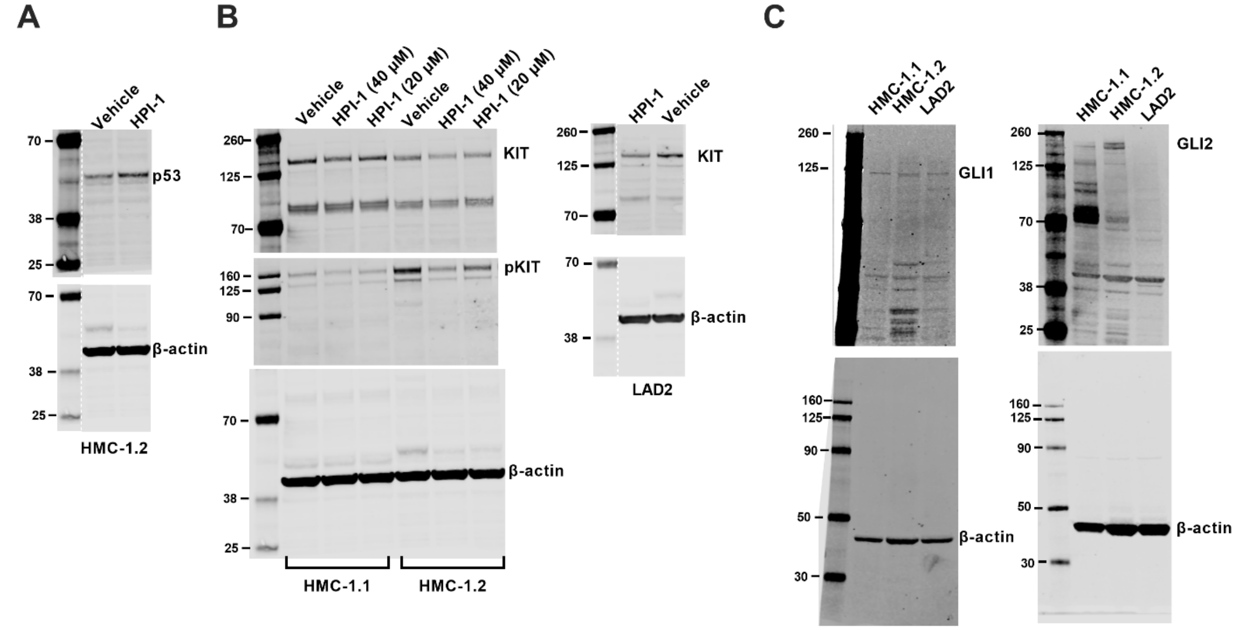

Supplementary Figure 6. Full scan of blots showed in Figure 6 (**A**), Figure 7 (**B**), and Supplementary Figure 1 (**C**). Note a major band of around 80KDa molecular size with the anti-GLI2 antibody, particularly in HMC-1.1 cells. This band does not represent repressor form of GLI2 since the antibody used does not recognize this form of GLI2 but may be a proteolytic fragment of GLI2. White dashed lines indicate non-consecutive lanes within the same membrane.
